# Supplementary material for: Patient Education Improves Pain and Health-Related Quality of Life in Patients with Established Spinal Osteoporosis in Primary Care—A Pilot Study of Short- and Long-Term Effects
Source: Int J Environ Res Public Health. 2023 Mar 10;20(6):4933. doi: 10.3390/ijerph20064933 (PMC10049553; doi:10.3390/ijerph20064933)
Supplement: Supplementary file 1 [file ijerph-20-04933-s001.zip › ijerph-2213608-supplementary.pdf]

Table S1. Questionnaires and clinical testing outcomes and change between baseline and post-observation.

	Baseline<br>Md [X] 25-75%	Post-Observation<br>Md [X] 25-75%	p-value
**RAND-36**			
Physical function PF	70 [60] 35-85	70 [59] 35-80	0.435
Role Physical RP	25 [39] 0-75	50 [50] 0-100	0.485
Bodily Pain BP	45 [49] 45-63	45 [51] 45-68	0.609
General Health GH	55 [52] 28-73	60 [56] 35-78	0.158
Vitality VT	55 [55] 33-73	65 [56] 38-78	0.677
Social Function SF	75 [75] 50-100	75 [72] 50-100	0.108
Role Emotional RE	100 [65] 33-100	100 [73] 33-100	0.491
Mental Health MH	76 [73] 66-88	84 [75] 58-92	0.366
**Qualeffo-41**			
Pain	55 [51] 35-65	40 [49] 33-70	0.327
Activities of Daily Life	13 [19] 6-25	19 [21] 13-28	0.323
Jobs Around the House	20 [27] 10-45	25 [28] 7,5-40	0.980
Mobility	22 [25] 9-46	19 [25] 11-39	0.878
Social Function	40 [41] 22-61	33 [40] 23-64	0.629
General Health Perception	58 [56] 38-71	58 [54] 33-75	0.530
Mental Function	36 [39] 31-50	36 [38] 26-50	0.923
Total score	32 [36] 25-48	28 [36] 25-48	0.602
**EQ-5D**			
EQ-5D Index	0.73 [0.63] 0.62-0.80	0.73 [0.66] 0.62-0.80	0.646
**Pain**			
Current pain, NRS	3.5 [3.2] 0.3-5.3	1.0 [1.7] 0.0-3.0	**0.019**
Pain last week, NRS	5.0 [5.2] 4.0-6.9	5.0 [5.0] 4.0-6.0	0.794
Worst pain, NRS	7.8 [7.3] 6.3-8.3	8.0 [7.2] 5.0-9.0	0.805
**Fall**			
FES-I	21 [26] 19-34	21 [26] 19-31	0.627
**Physical activity**			
Physical exercise weekly (min)	30 [42] 8-75	30 [41] 0-75	0.743
Everyday activity weekly (min)	225 [198] 98-300	225 [215] 150-300	0.196
Total Physical activity weekly (min)	255 [235] 116-323	300 [256] 150-345	0.348
Daily sitting/resting (hours)	5 [5] 2-7	5 [5] 2-7	0.85

**Clinical tests**			
Distance C7-wall (cm)	6.5 [7.9] 5-11	6 [7] 5-9	**0.025**
Hand force right	20 [20] 17-24	20 [21] 17-26	0.338
Hand force left	18 [19] 14-23	17 [19] 15-23	0.691
Chair-stand test	9 [9] 7-11	11 [12] 10-14	**0.002**
Right leg, eyes open (s)	30 [23] 15-30	20 [19] 8-30	0.32
Left leg, eyes open (s)	27 [21] 12-30	29 [21] 9-30	0.674
Right leg, eyes closed (s)	3 [4] 2-5	3 [3] 2-4	0.931
Left leg, eyes closed (s)	2 [3] 1-4	3 [4] 1-5	0.114
Walking forwards (steps)	15 [12] 10-15	15 [12] 10-15	0.953
Walking backwards (steps)	15 [11] 5-15	15 [11] 2-15	0.465
Weight (kg)	63.8 [66.7] 55.8-74.4	63 [66.7] 55.0-76.0	0.972
Height (cm)	160 [161] 154-169	160 [161] 153-169	0.275

Abbreviations: Md, Median; [X], Mean; Bold text: $p < 0.05$. All n=21
